# Molecular characteristics of *Staphylococcus aureus* strains isolated from subclinical mastitis of water buffaloes in Guangdong Province, China

**DOI:** 10.3389/fvets.2023.1177302

**Published:** 2023-11-08

**Authors:** Dexian Zhang, Ximing Lu, Xiangyan Feng, Xuzeng Shang, Qingyou Liu, Nan Zhang, Hong Yang

**Affiliations:** ^1^School of Life Science and Engineering, Foshan University, Foshan, China; ^2^Liaoning Agricultural Development Service Center, Shenyang, China

**Keywords:** *Staphylococcus aureus*, antimicrobial resistance, virulence genes, biofilm formation, subclinical mastitis in water buffaloes

## Abstract

Intramammary infections (IMI) in animals reared for milk production can result in large economic losses and distress to the animals. *Staphylococcus aureus* is an important causative agent of IMI in dairy cows, but its prevalence in water buffaloes has not been determined. Therefore, the current study was conducted to investigate the prevalence of subclinical mastitis in water buffaloes and the antimicrobial susceptibility, virulence genes and biofilm formation abilities of *Staphylococcus aureus* isolates recovered from water buffaloes in Guangdong, China. *Staphylococcus aureus* strains were isolated from milk samples of water buffaloes with subclinical mastitis, and twofold microdilution, PCR and crystal violet staining methods were used to determine antimicrobial susceptibility, distributions of virulence and antimicrobial resistance genes and biofilm formation ability, respectively. Our results indicated that 29.44% of water buffaloes were diagnosed with subclinical mastitis, and the most prevalent pathogens were *Escherichia coli* (96.17%), coagulase-negative staphylococci (CoNS) (67.60%) and *S. aureus* (28.57%). Most *S. aureus* isolates showed resistance to bacitracin, doxycycline, penicillin, florfenicol, and tetracycline but were susceptible to ciprofloxacin, ceftizoxime, cefoquinoxime, and ofloxacin. Moreover, 63.72% of *S. aureus* isolates were positive for *tetM,* and the prevalence of *msrB*, *blaZ*, *mecA*, *fexA*, and *tetK* ranged from 21.24 to 6.19%. All *S. aureus* isolates harbored *clfB* and *icaA* genes, and the virulence genes *hla* (93.8%), *hld* (91.15%), *clfA* (90.27%), *fnbA* (86.73%), and *hlb* (83.19%), and *tsst*, *icaD*, *sec*, *see*, *fnbB*, and *sea* showed a varied prevalence ranging from 3.5 to 65.49%. All *S. aureus* isolates possessed the ability to form biofilms, and 30.09% of isolates showed strong biofilm formation abilities, while 19.47% of isolates were weak biofilm producers. Our results indicated that subclinical mastitis is prevalent in water buffaloes in Guangdong, China, and *S. aureus* is prevalent in samples from water buffaloes with subclinical mastitis. Most *S. aureus* isolates were susceptible to cephalosporins and fluoroquinolones; thus, ceftizoxime and cefoquinoxime can be used to treat subclinical mastitis in water buffaloes.

## Introduction

Water buffalo (*Bubalus bubalis*) is of importance in the milk industry and contributes to approximately 15% of milk production (1). Many parts of the world have traditionally produced buffalo milk, including Asia, Egypt, and Europe. China produces approximately 5% of global buffalo milk, and Guangdong, Guangxi and Hunan are its primary producers (2). Italy is the main producer of buffalo milk in Europe because of the popularity of buffalo mozzarella cheese (3), which retails for twice the price of bovine milk cheese (4).

Mastitis is one of the most prevalent diseases among dairy animals, causing economic losses to milk producers due to a decrease in milk quality and production, an increase in veterinary and labor costs and an increased rate of culling (5). Bacteria are the primary causative agents of mastitis, although physical trauma and mechanical injury also contribute (1). The primary bacterial causes of mastitis in both dairy cows and water buffaloes are *Staphylococcus aureus*, *Streptococcus agalactiae*, *Streptococcus dysgalactiae*, and *Escherichia coli*. *Staphylococcus aureus* is one of the primary pathogens causing mastitis in dairy cows at a level of approximately 40% in China (6).

Among antimicrobial agents, antibiotics are normally used to treat infections caused by bacteria, including intramammary infections (IMI) in domestic animals. In dairy animals, mastitis and reproductive diseases often require prolonged use of antimicrobial agents (7). Unfortunately, the widespread use of antimicrobial agents has resulted in high levels of antimicrobial resistance, leading to clinical treatment failures. Therefore, monitoring systems for antimicrobial resistance of bacterial pathogens can provide essential information for the rational use of antimicrobial agents when treating infections (8). Knowledge regarding the prevalence of mastitis and the knowledge of pathogens causing mastitis is critical in preventing the occurrence of mastitis and can provide effective measures for control and appropriate therapeutic protocols (9).

There are two groups of virulence genes in *S. aureus,* including surface-localized structural components serving as virulence factors and secreted virulence factors involved in evading host defenses and colonizing mammary glands (10). Surface-localized structural components include membrane-bound factors (fibrinogen binding protein, collagen binding protein and elastin binding protein), cell wall-bound factors (lipoteichoic acid, peptidoglycan, protein A and protease) and cell surface-associated factors (capsule). Secretory factors include toxins (staphylococcal enterotoxins, leucocidin, toxic shock syndrome toxin, and hemolysins) and enzymes (staphylokinase, coagulase, lipase, DNase, and hyaluronidase). Moreover, biofilm formation also contributes to adhesion and invasion into mammary epithelial cells and thus provides an escape from the host immune system. Enterotoxins often lead to food poisoning and include staphylococcal enterotoxins A to F and G to Q (11). Enterotoxins G to Q are more prevalent in isolates from dairy cows with mastitis than in isolates from cows without mastitis; this has implicated these virulence factors in the occurrence of mastitis (12). However, the clear mechanisms of virulence in mastitis of dairy cows need further study.

In China, the number of water buffalo farms is increasing, and thus, mastitis is occurring more frequently in these animals. Mastitis can be divided into two forms: clinical and subclinical. In clinical mastitis, clots and flakes can be observed in milk, and the quarters become swollen with severe conditions leading to the formation of lacerations, necrosis and cord formation of the teat. While no clinical signs or symptoms can be seen in subclinical mastitis, there is a reduction in milk production and deterioration of milk quality (13). Subclinical infection is 15–40 times more prevalent than clinical infection and can rapidly spread on a farm (14).

To reduce the prevalence of subclinical mastitis in water buffaloes, it is essential to have knowledge of prevalence data and understand the antimicrobial susceptibility profiles of subclinical mastitis caused by *S. aureus*. Therefore, this study was conducted to investigate the prevalence of subclinical mastitis in water buffaloes in Guangdong, China, and to determine the antimicrobial susceptibility of *S. aureus* isolates and their antimicrobial resistance and virulence determinants.

## Materials and methods

### Sample size

Ausvet epidemiological calculators^1^ were used to calculate the sample sizes according to the method from Charan and Biswas at a 95% confidence level (15) as follows:


$$\mathrm{Sample size}=\frac{Z_{1-\frac{\alpha }{2}}^{2}P\left(1-p\right)}{d^{2}}$$

where $z_{1-\alpha /2}$ equals 2.58 with a 1% type error (*p* < 0.01). The expected proportion of water buffaloes in Guangdong Province is 0.05, and the expected precision (d) is 0.01.

A total of 3,900 samples from 975 water buffaloes were included in this study from the following regions: Qingyuan (*n* = 884), Guangzhou (*n* = 1,076), Jiangmen (*n* = 992) and Zhaoqing (*n* = 948). The average sample number per farm was 342.9 (range 174 to 584).

### Sample collection

The milking of animals on all farms was performed twice a day, and the sample collection procedure consisted of fore-stripping (3–5 squirts of milk) followed by teat disinfection with 0.25% iodine solution and drying with a clean towel. The milking clusters were then attached and removed automatically when finished, followed by postmilking teat disinfection with 0.5% iodine. Duplicate milk samples for each quarter were aseptically collected according to standard protocols of the National Mastitis Council (16). Briefly, milk samples (3 mL) were collected from all quarters of each animal after the first 3 streams of milk were discarded and placed in an ice box and transferred to the laboratory within 6 h. Presumptive evidence of subclinical mastitis (17) was determined using a commercial California Mastitis Test kit (CMT) (ImmuCell, Portland, ME, United States) following the recommendations of the manufacturer. Briefly, 2 mL of milk sample was mixed with an equal amount of CMT solution in the paddle and stirred for 30 s. Thickening indicated elevated somatic cell counts (SCC), and these samples were then used for bacterial isolation and identification.

### Bacterial isolation

Identification of *S. aureus* from milk samples was carried out as previously described (18). Briefly, a 0.1 mL milk sample was inoculated into 3 mL tryptic soy broth (TSB; AoBox, Beijing, China) containing 10% NaCl and incubated at 37°C for 24 h. A loopful of enrichment broth culture was streaked onto a Baird-Parker agar plate (AoBox) and incubated at 37°C for 24 h. The suspected *S. aureus* colonies had a black and shiny appearance with a thin white border surrounded by a light area. The suspected colonies were streaked onto chromogenic *S. aureus* agar plates (CHROMagar, Paris, France). At least 3 positive colonies per sample were then confirmed as coagulase-positive *S. aureus* using commercial API STAPH test strips (bioMérieux, Marcy-l’Ѐtoile, France). *Staphylococcus aureus* isolates were stored at −80°C in cryogenic vials (Biologix, Shandong, China) containing 1 mL TSB and 30% glycerin. Coagulase-negative staphylococci were isolated as previously described (19). In brief, milk samples were cultured on blood agar plates (AoBox) at 37°C for 48 h. Typical colonies were selected and identified using classical biochemical methods, including Gram staining and oxidase, catalase and DNase tests, and the ability to coagulate rabbit plasma using commercial kits (Sigma Chemical, Shanghai, China). If the suspected strains failed biochemical identification, molecular identification using PCR amplification and sequencing of the *sodA* gene was performed as previously described (19).

*Escherichia coli* isolation utilized a 0.1 mL milk sample inoculated in 3 mL Mueller-Hinton broth (MHB; Aobox), which was incubated at 37°C for 24 h. Samples were then streaked onto MacConkey Agar plates, and plates were kept at 37°C for 24 h. Presumptive identification of *E. coli* were pink colonies that were then subjected to matrix assisted laser desorption ionization-time of flight mass spectrometry (MALDI-TOF MS) using a Microflex LT instrument (Bruker Daltonics, Bremen, Germany).

*Streptococcus agalactiae* and *S. dysgalactiae* were identified as previously described (20) using EN medium, and positive isolates were then transferred to Columbia Blood Agar Base Medium containing 5% sheep blood (Hope Bio-Technology, Qingdao, China) and incubated at 37°C for 24 h. Colonies with typical *Streptococcus* morphologies were then subjected to catalase and 6.5% NaCl tests. Isolates were further identified as *S. agalactiae* and *S. dysgalactiae* according to reactions using the sodium hippurate test, esculin hydrate, and CAMP tests.

### Antimicrobial susceptibility testing

Antimicrobial resistance phenotypes of *S. aureus* isolates were determined using the microdilution method in MH broth according to the Clinical Laboratory and Standards Institute guidelines (CLSI) (21). A loopful of each *S. aureus* isolate preserved in glycerinated TSB was streaked on a Baird-Parker agar plate and incubated at 37°C for 24 h. The colonies were inoculated in MH broth, and the cultures were diluted in sterile normal saline and adjusted to a turbidity of 0.5 McFarland standard (10^5^–10^6^ colony-forming units (CFU)/mL). The suspension was then swabbed on Muller-Hinton agar plates and incubated at 37°C for 24 h as previously described. *Staphylococcus aureus* ATCC 25923 was used as the reference strain. Each experiment was repeated at least three times. Breakpoints for different antimicrobial agents were based on CLSI guidelines (21).

Antimicrobial agents included ceftiofur (CTF) (0.02–16 μg/mL), cefoquinoxime (CFQ, 0.03–32 μg/mL), ceftizoxime (CFT, 0.03–32 μg/mL), cefoxitin (CFX, 0.03–32 μg/mL), florfenicol (FLO, 0.125–128 μg/mL), ciprofloxacin (CIP, 0.03–32 μg/mL), enrofloxacin (ENO, 0.03–32 μg/mL), ofloxaxin (OFX, 0.06–64 μg/mL), erythromycin (ERY, 0.125–128 μg/mL), azithromycin (ATM, 0.125–128 μg/mL), gentamycin (GEN, 0.125–128 μg/mL), penicillin (PEN, 0.03–32 μg/mL), ampicillin (AMP, 0.125–128 μg/mL), tetracycline (TET, 0.125–128 μg/mL), doxycycline (DXC, 0.03–32 μg/mL) and bacitracin (BTC, 4–1024 μg/mL).

### Antimicrobial resistance and virulence gene detection

Strains used for testing were taken from frozen stocks, cultures were streaked onto TSA plates containing 5% sterile defibrinated sheep blood, and the plates were incubated at 37°C for 24 h. Single colonies were inoculated into 3 mL TSB and cultured with shaking at 37°C for 24 h. The cultures were centrifuged at 3000 × *g,* and cell pellets were suspended in phosphate buffered saline (PBS, pH 7.4; Solarbio, Beijing, China) containing 20 mg/mL lysostaphin (Meilunbio, Dalian, China). The mixture was kept at 37°C for 30 min, genomic DNA was extracted using a TIANamp bacterial DNA extraction kit (TianGen, Beijing, China), and DNA quality was evaluated by UV spectroscopy with a NanoDrop-2000 instrument (Thermo Fisher, Shanghai, China). The extracted DNA was diluted to 50 mg/L in sterile deionized water for PCR assays (see below).

Antimicrobial resistance genes (ARGs) were detected using multiplex PCR as previously described (22). Briefly, PCRs included gene-specific primers for the following ARG groups: penicillin (*blaZ*), macrolide (*msrA* and *msrB*), erythromycin (*ermA* and *ermC*), streptogramin acetyltransferase genes (*vatA*, *vatB*, and *vatC*), aminoglycoside (*aacA-D*), tetracycline (*tetK* and *tetM*), lincosamide (*linA*), methicillin (*mecA*), florfenicol (*fexA*), oxazolidine ketone (*cfr* and *optrA*) and vancomycin (*vgaA* and *vgaC*).

Virulence genes (*hla*, *hlb*, *hld*, *sea*, *seb*, *sec*, *sed*, *see*, *tst*, and *lukF*), biofilm-related genes (*bap*, *icaA*, and *icaD*) and adhesion-related genes (*fnbA*, *fnbB*, *clfA*, and *clfB*) were detected using PCR as previously described (23). The primers were provided by Sangon Biotech (Shanghai, China), and water rather than DNA template was added as a contamination control. DNA from isolates that harbored virulence genes or ARGs was used as a positive control. These were included in all PCRs. Gene amplifications were performed using a commercial PCR instrument (Bio-Rad, Hercules, CA, United States) as previously described (24). Briefly, the PCR mixture contained DNA (1 μL), 0.2 μL of each primer, Prime STAR Max DNA polymerase (12.5 μL), and ddH_2_O (11.1 μL). The PCR cycling conditions consisted of an initial denaturation at 95°C for 10 min, followed by 30 cycles of 95°C for 30 s, annealing at appropriate temperatures for 30 s (Supplementary Table S1) and extension at 72°C for 1 min.

### Biofilm formation

Biofilm formation was determined in 96-well microtiter plate assays using minimal medium M9 (Sigma Chemical) as previously described (25). Briefly, the overnight cultures in TSB were diluted 1:100, and 200 μL was transferred into each well of the microtiter plate that was incubated at 37°C for 72 h. Each well was washed with 200 μL PBS after the supernatant was discarded and fixed with 200 μL methanol for 20 min and washed again with PBS 3 × and then stained with 0.4% crystal violet (Meilunbio, Dalian, China) for 15 min. The biofilms were then dissolved in 200 μL 33% (w/v) acetic acid for 30 min. The biofilm formation was measured at 590 nm optical density (OD_590 nm_) in a Bio-Rad plate reader (Shanghai, China). The strong biofilm-forming strain *Salmonella enterica* Typhimurium ATCC 14028 was used as the positive control, and sterile TSB was used as the negative control for the biofilm formation assay (26). All assays were performed in triplicate. The OD_590 nm_ value of 0.6 was applied as the cutoff point to distinguish between biofilm producers and nonbiofilm producers (10). Biofilm formation was classified as strong +++ (OD_590 nm_ > 1.8), moderate ++ (1.8 > OD_590 nm_ > 1.2), weak + (1.2 > OD_590 nm_ > 0.6), and negative − (OD_590 nm_ < 0.6).

### Statistical analysis

Pearson correlation analysis was applied to differences in antimicrobial resistance rates in correlation to antimicrobial resistance genes harbored by *S. aureus* isolates. *T* tests were used to analyze the significance of biofilm formation between *S. aureus* isolates. All analyses were carried out using Prism 8 (GraphPad, Boston, MA, United States).

## Results

### Prevalence of subclinical mastitis in water buffaloes

Our screening of 975 water buffaloes indicated that 287 (29.44%) were considered to have subclinical mastitis according to the CMT tests. Strongly positive (+++) results were observed in 53.31% (153/287) of the cases, while mild and moderate intensity results occurred in 26.13% (75/287) and 20.56% (59/287) of the cases, respectively. *Escherichia coli* (276/287) was the most common bacteria isolated from these positive samples, followed by coagulase-negative staphylococci (CNS) (194/287). *Staphylococcus aureus* (113/287), *S. agalactiae* (82/287) and *S. dysgalactiae* (41/287).

### Antimicrobial resistance phenotype

Resistance to bacitracin, doxycycline, penicillin, florfenicol and tetracycline was observed in 90.27, 84.07, 84.96, 81.42 and 82.3% of the examined *S. aureus* isolates, respectively (Table 1). A lower prevalence of resistance was noted for ciprofloxacin (7.08%), ceftizoxime (15.04%), cefoquinoxime (18.58%) and ofloxacin (16.81%). Among *S. aureus* isolates, only 12 (10.62%) were resistant to cefoxitin, and these were classified as phenotypic methicillin-resistant *S. aureus* (MRSA).

**Table 1:** Antimicrobial susceptibility of *S. aureus* isolates from subclinical mastitis of buffaloes.

Antimicrobial agents	MICs (μg/mL)			Break point	Resistance	Mediate	Susceptibility
	MIC50	MIC90	Range				
Penicillin	4	32	0.03– ≥ 128	≤0.12, –, ≥0.25	84.96% (96/113)		15.04% (17/113)
Ampicillin	0.125	16	0.125– ≥ 128	≤0.25, –, ≥ 0.5	55.75% (63/113)		54.25% (50/113)
Cefoquinoxime	0.06	16	0.06–32	≤2, 4, ≥ 8	18.58% (21/113)	12.39% (14/113)	69.03% (78/113)
Ceftizoxime	0.12	8	0.12–32	≤2, 4, ≥ 8	15.04% (17/113)	10.62% (12/113)	74.34% (84/113)
Ceftiofur	4	8	0.125– ≥ 128	≤2, 4, ≥ 8	27.43% (31/113)	36.28% (41/113)	36.28% (41/113)
Cefoxitin	0.012	16	0.006–32	≤4, −, ≥8	10.62% (12/113)	0	89.38% (101/113)
Chloramphenicol	16	≥128	0.5– ≥ 128	≤4, 8, ≥ 16	81.42% (92/113)	10.62% (12/113)	7.96% (9/113)
Ciprofloxacin	0.5	4	0.125–64	≤1, 2, ≥ 4	7.08% (8/113)	15.04% (17/113)	77.88% (88/113)
Ofloxacin	0.5	32	0.06–64	≤1, 2–4, ≥ 8	16.81% (19/113)	19.47% (22/113)	63.72% (72/113)
Enrofloxacin	1	32	0.06– ≥ 128	≤0.5, 1–2, ≥ 4	37.17% (42/113)	20.35% (23/113)	42.48% (48/113)
Erythromycin	≥128	≥128	0.125– ≥ 128	≤0.5, 1–4, ≥ 8	74.34% (84/113)	7.08% (8/113)	18.58% (21/113)
**Azithromycin**	4	≥128	0.06– ≥ 128	≤2, 4, ≥ 8	32.74% (37/113)	19.47% (22/113)	47.79% (54/113)
Gentamicin	1	32	0.125– ≥ 128	≤4, 8, ≥ 16	21.24% (24/113)	15.04% (17/113)	63.72% (72/113)
Tetracycline	8	≥128	0.125– ≥ 128	≤0.25, 0.5, ≥ 1	82.3% (93/113)	2.65% (3/113)	15.04% (17/113)
Doxycycline	8	32	0.125–32	≤0.12, 0.25, ≥ 0.5	84.07% (95/113)	3.54% (4/113)	12.39% (14/113)
Bacitracin	256	512	4– ≥ 1,024	≤64, 128, ≥ 256	90.27% (102/113)	3.54% (4/113)	6.19% (7/113)

### Distribution of antimicrobial resistance genes among *Staphylococcus aureus* isolates

**Figure 1:**
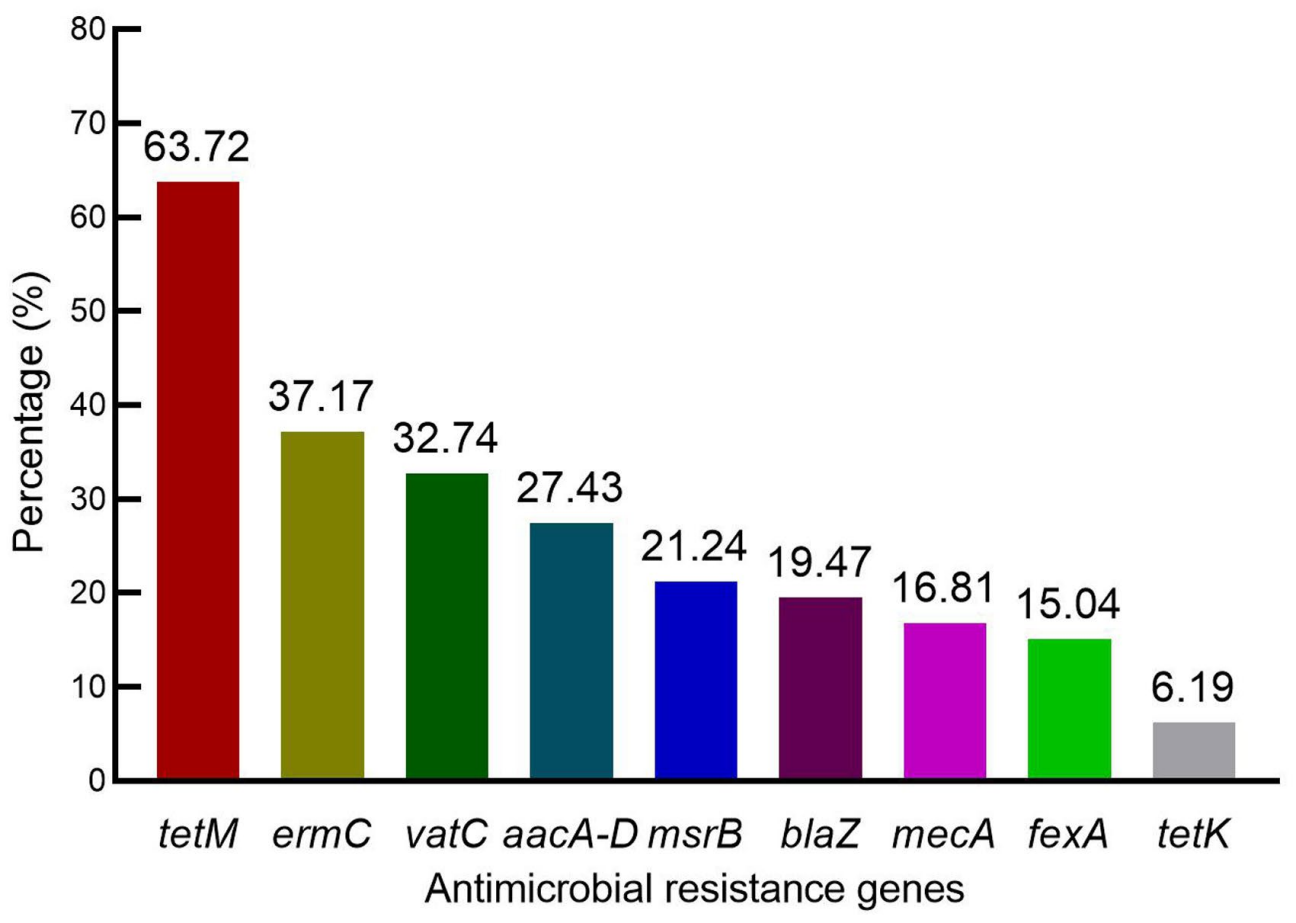
Distribution of antimicrobial resistance genes among *S. aureus* isolates.

The ARGs possessed by *S. aureus* isolates included *tetM*, *ermC*, *vatC*, and *aacA-D* in 63.72% (72/113), 37.17% (42/113), 32.74% (37/113), and 27.43% (31/113), respectively. Moreover, the prevalence of *msrB*, *blaZ*, *mecA*, *fexA*, and *tetK* was 21.24, 19.47, 16.81, 15.04, and 6.19%, respectively (Figure 1). Interestingly, ARGs for macrolide resistance *msrA*, erythromycin resistance *ermA*, streptogramin resistance *vatA* and *vatB,* oxazolidinone resistance *cfr* and *optrA* and vancomycin resistance *vgaA* and *vgaC* were not detected. These results indicated a lack of a correlation between resistance phenotypes and ARG distributions among the *S. aureus* isolates.

### Prevalence of virulence-associated genes

**Figure 2:**
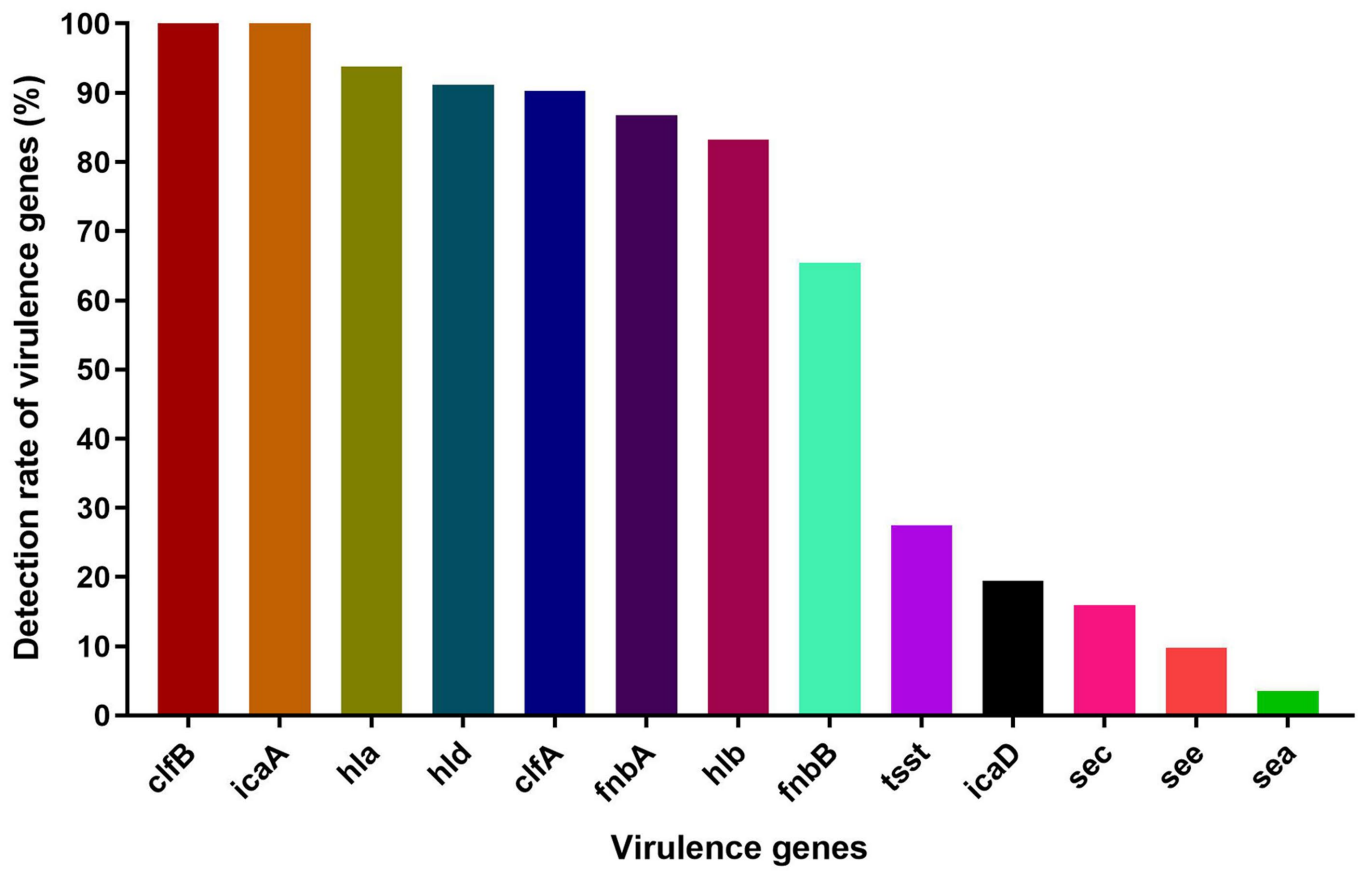
The detection rate of virulence genes among *S. aureus* isolates.

The virulence-associated genes we detected in this study were distributed with varying frequencies among *S. aureus* isolates (*n* = 113) (Figure 2). In particular, *clfB* and *icaA* were present in all *S. aureus* isolates, and nearly all harbored *hla* (93.8%), *hld* (91.15%), *clfA* (90.27%), *fnbA* (86.73%) and *hlb* (83.19%). In contrast, a lower prevalence was found for *tsst* (27.43%), *icaD* (19.47%), *sec* (15.93%), *see* (9.73%), *fnbB* (65.49%) and *sea* (3.54%). The virulence genes *seb*, *sed*, *bap*, and *lukF* were not detected in any isolates.

### Biofilm formation ability

**Figure 3:**
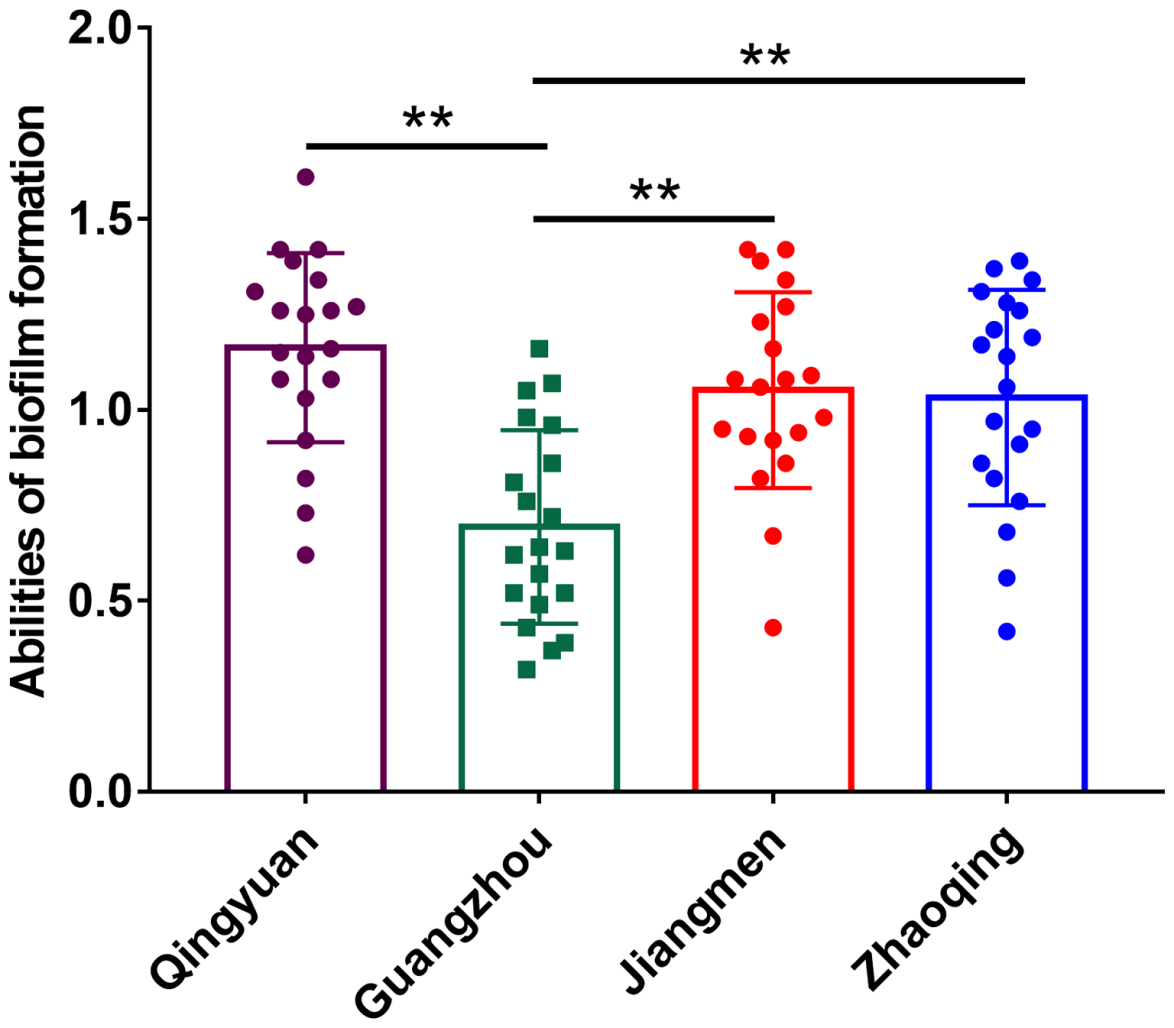
Biofilm formation of *S. aureus* isolates in different areas of Guangdong. Note: There were no significant difference between *S. aureus* isolates from Qingyuan, Jiangmen, and Zhaoqing. But *S. aureus* isolates from Guangzhou were significantly lower (*p* < 0.01) in comparison with isolates from Qingyuan, Jiangmen, and Zhaoqing. ^**^ means *p* < 0.01.

All our milk samples produced isolates able to form biofilms. The rates of strong, moderate and weak biofilm producers were 30.09, 50.44, and 19.47%, respectively. In particular, most Qingyuan isolates (70.83%, 17/24) were strong biofilm producers, while 20.83% displayed moderate phenotypes. In contrast, only 9.76% (4/41) of the Guangzhou isolates were strong biofilm producers, and 65.85% (27/41) were moderate producers. Biofilm formation in *S. aureus* isolates from Guangzhou, Qingyuan, Jiangmen and Zhaoqing was 0.69 ± 0.24, 1.16 ± 0.35, 1.05 ± 0.25, and 1.03 ± 0.28, respectively. The biofilm formation of isolates from Guangzhou was significantly lower (*p* < 0.01) than that of isolates from other areas (Figure 3; Table 2).

**Table 2:** Biofilm formation ability of *S. aureus* isolates from water buffaloes.

Areas	Strong biofilm formation	Mediate biofilm formation	Weak biofilm formation
Qingyuan	70.83% (17/24)	16.67% (4/24)	12.5% (3/24)
Guangzhou	9.76% (4/41)	65.85% (27/41)	24.39% (10/41)
Jiangmen	18.52% (5/27)	51.85% (14/27)	25.93% (7/27)
Zhaoqing	38.1% (8/21)	42.86% (9/21)	16.67% (4/21)
Average	30.09% (34/113)	50.44% (57/113)	19.47% (22/113)

## Discussion

Mastitis is a disease that is globally prevalent in dairy animals (1), and water buffaloes are generally less susceptible to this infection in comparison with dairy cows because of strong muscles at the opening of the teat canal (27). In the current study, 29.44% of water buffaloes were diagnosed with subclinical mastitis, which was consistent with a previous report for these animals (28). Previous studies have indicated that the prevalence of subclinical mastitis in water buffaloes ranges from 6.0 to 87% (27). It seems that factors such as animal age, stage of lactation, management style and farm environment may have contributed to these variations (13). To our knowledge, few studies have been carried out to investigate the bacteriology of subclinical mastitis in water buffaloes. In this study, we found that CoNS, *E. coli* and *S. aureus* were the dominant bacterial pathogens and that *S. agalactiae* and *S. dysgalactiae* were present to a lesser degree. *Escherichia coli* infections often lead to severe systemic clinical symptoms, and this was the most prevalent pathogen in our study. Similarly, *E. coli* was the most prevalent pathogen in subclinical mastitis infections in a Nepal water buffalo study (28), while *Streptococcus* (39.2%) was the most prevalent pathogen in mastitis dairy cows, and only 8.4% were present as *E. coli* (29). Therefore, it seems that other factors (see above) may influence the occurrence of mastitis. Moreover, *S. aureus* (61%) was the dominant pathogen in cattle from Jammu and Kashmir with mastitis, and *E. coli* (13%), CoNS (13.04%), *Streptococcus uberis* (4.35%) and *S. dysgalactiae* (8.69%) were also isolated from samples (30). Similar results were reported by other researchers (27); the reasons for this might be topographical and management conditions and the difference between dairy cows and water buffaloes.

Antimicrobial susceptibility can provide important information in choosing antimicrobial agents when treating infections. In this study, *S. aureus* isolates showed high resistance to penicillin, florfenicol, erythromycin, tetracycline, doxycycline and bacitracin. Similar results were observed in *S. aureus* isolates from dairy cows with mastitis in northern China (31). However, other researchers reported lower rates of antimicrobial resistance in *S. aureus* isolates in Pakistan (13). Cephalosporins are important antimicrobial agents, and *S. aureus* isolates resistant to ceftiofur have been reported (23). Similarly, antimicrobial resistance to cephalosporins, including cefoquinoxime, ceftizoxime, and ceftiofur, was detected in our study. β-lactams, fluoroquinolones, and aminoglycosides are commonly used to treat dairy mastitis (31), and this most likely contributed to the high levels of resistance we found to these agents. Saini and his colleagues also found that the herd level of antimicrobial agents used when treating mastitis in bovines was positively correlated with antimicrobial resistance among isolates from mastitic animals (32). Unfortunately, the use of antimicrobial agents in these farms was not documented in our study, so we cannot directly correlate the use of antimicrobial agents and antimicrobial resistance. Moreover, we detected antimicrobial resistance genes among isolates, although we found no significant correlation between phenotype and genotype. For example, only 22.92% (22/96) of isolates resistant to penicillin carried the *blaZ* gene, similar to previous findings (33, 34). These inconsistencies indicated that the presence of a particular ARG was not an indicator of phenotypic resistance, and this can be influenced by numerous genetic and environmental conditions (35).

MRSA is a global health concern since it is not only resistant to β-lactams but also nonsusceptible to other commonly used antimicrobial agents (36). In the Philippines and Pakistan, the MRSA prevalence was 25.81 and 19.6%, respectively, in water buffaloes with mastitis (13, 37), while a much lower rate (2.2%) of MRSA was detected in water buffaloes with mastitis in Iran (38). Several factors, such as age, feeding status, body conditions, and hand or machine hygiene on the farm, may contribute to this phenomenon. Several technologies, such as nanoparticles and antibiotics combined with plant extracts or microparticles, are widely used in food, veterinary and animal science. For example, a report indicated that antibiotics coupled with zinc oxide nanoparticles can significantly increase the zone of inhibition; similarly, amoxicillin showed the highest increase in inhibitory effects against MRSA when combined with *Calotropis procera* extract (39). These technologies are believed to be promising methods for treating infections caused by MRSA.

Biofilms can increase the resistance of *S. aureus* to antimicrobial agents and are responsible for persistent infections (40). Biofilms are composed of multiple layers of bacteria, which prevents the permeability of antimicrobial agents and thus increases tolerance. In our study, we investigated the biofilm formation ability of *S. aureus* isolates grouped by area. Interestingly, *S. aureus* isolates from Guangzhou showed significantly lower levels of biofilm formation in comparison with isolates from other areas (*p* < 0.01). However, the antimicrobial resistance of *S. aureus* isolates did not differ by region (data not shown). Similarly, a previous study indicated that the biofilm formation ability of ST7 and ST188 strains was much higher than that of other lineages even though their phenotypic antimicrobial resistance was comparable with that of other lineages (41). These data indicated that gene mutations, horizontal gene transfer and modifications of antibiotic molecules are the primary modes of antimicrobial resistance in *S. aureus* isolates from water buffaloes and that biofilm formation plays only a secondary role (42).

Virulence genes contribute to the pathogenesis of *S. aureus* infections. Adhesion is the first step for *S. aureus* to invade host cells and immune responses (43) and involves *clfA*, *clfB*, *fnbA*, and *fnbB*. In our study, all isolates carried *clfB,* and most isolates carried *clfA*, *fnbA*, and *fnbB*. These results were similar to previous reports where the *clfB* gene was detected in all isolates from bovine mastitis samples, and *fnbA* and *clfB* were comparable with the levels we found (44, 45). In contrast, much lower detection levels were reported for *fnbB* in *S. aureus* isolates from Algeria and Australia (43, 46).

Hemolysins are also involved in invasion and the host immune response (44, 46). In our study, over 80% of our total isolates carried *hla*, *hlb*, and *hld,* consistent with previous reports (41, 44). Toxic shock syndrome toxin, a superantigen encoded by the *tsst* gene, can lead to toxic shock syndrome in humans (47). and the *tsst* prevalence in *S. aureus* isolates ranged from 2.1 to 40.0% (10, 44) and was 25% in our study. It is therefore important to monitor the epidemiology of such super antigenic toxin genes to protect public health from this threat.

## Conclusion

In conclusion, subclinical mastitis was prevalent among water buffaloes in Guangdong, China, and *S. aureus* was identified as a significant pathogen associated with subclinical mastitis of water buffaloes. The majority of *S. aureus* isolates exhibited resistance against bacitracin, doxycycline, penicillin, florfenicol, and tetracycline while maintaining susceptibility to other antimicrobial agents, including ciprofloxacin, ceftizoxime, cefoquinoxime, and ofloxacin. Furthermore, the *S. aureus* isolates harbored various virulence genes, such as *hla*, *hld*, *clfA*, *fnbA*, and *hlb*. Notably, all *S. aureus* isolates showed the ability to form biofilms, with nearly one-third of the isolates possessing strong biofilm formation abilities. Given these findings, antibiotics should be cautiously used when treating subclinical mastitis in water buffaloes within this region. Additionally, the impact of biofilm formation on the transmission of antibiotic resistance must be investigated in further studies.

## Data availability statement

The raw data supporting the conclusions of this article will be made available by the authors, without undue reservation.

## Ethics statement

The animal studies were approved by the Ethical Committee of Foshan University. The studies were conducted in accordance with the local legislation and institutional requirements. Written informed consent was obtained from the owners for the participation of their animals in this study.

## Author contributions

DZ, NZ, and HY: conceptualization and writing – review and editing. XL and XS: methodology and data curation. XF and QL: analysis. DZ: writing original draft preparation and funding acquisition. HY: supervision and project administration. All authors contributed to the article and approved the submitted version.

## Footnotes

^1^https://ausvet.com.au/
