# Supplementary material for: Molecular characteristics of Staphylococcus aureus strains isolated from subclinical mastitis of water buffaloes in Guangdong Province, China
Source: Front Vet Sci. 2023 Nov 8;10:1177302. doi: 10.3389/fvets.2023.1177302 (PMC10663324; doi:10.3389/fvets.2023.1177302)
Supplement: Supplementary file 1 [file Table_1.DOCX]

Supplementary Material

**Molecular characteristics of *Staphylococcus aureus* strains isolated form subclinical mastitis of water buffaloes in Guangdong Province, China**

Dexian Zhang^1^, Ximing Lu^1^, Xiangyan Feng^1^, Xuzeng Shang^2^, Qingyou Liu^1^, Nan Zhang^1^*, Hong Yang^1^*

*These authors contribute equally to this research

Corresponding Author:

Nan Zhang

Email: [zhangnan@fosu.edu.cn](mailto:zhangnan@fosu.edu.cn)

Tel: +86 (757)85505103

Hong Yang

Email: [yhong007@fosu.edu.cn](mailto:yhong007@fosu.edu.cn)

Tel: +86 (757)85505103

Table S1 Primers used in this research for detection of antimicrobial resistance genes and virulence genes

| Target genes |  | Sequence (5’-3’) | Amplicon size (bp) | Annealing temperature (°C) | References |
| --- | --- | --- | --- | --- | --- |
| *blaZ* | Forward | ATTTTGAAAAAGTTAATATTTTTAATTG | 700 | 55 | 41 |
|  | Reverse | CATTACACTCTTGGCGGTTTC |  |  |  |
| *msrA* | Forward | GGCACAATAAGAGTGTTTAAAGG | 940 | 50 | 42 |
|  | Reverse | AAGTTATATCATGAATAGATTGTCCTGTT |  |  |  |
| *msrB* | Forward | TATGATATCCATAATAATTATCCAATC | 595 | 50 | 42 |
|  | Reverse | AAGTTATATCATGAATAGATTGTCCTGTT |  |  |  |
| *ermA* | Forward | GTTCAAGAACAATCAATACAGAG | 421 | 52 | 42 |
|  | Reverse | GGATCAGGAAAAGGACATTTTAC |  |  |  |
| *ermB* | Forward | CCGTTTACGAAATTGGAACAGGTAAAGGGC | 359 | 55 | 42 |
|  | Reverse | GAATCGAGACTTGAGTGTGC |  |  |  |
| *vatA* | Forward | TGGTCCCGGAACAACATTTAT | 268 |  | 43 |
|  | Reverse | TCCACCGACAATAGAATAGGG |  |  |  |
| *vatB* | Forward | GCTGCGAATTCAGTTGTTACA | 136 | 58 | 42 |
|  | Reverse | CTGACCAATCCCACCATTTTA |  |  |  |
| *vatC* | Forward | AAGGCCCCAATCCAGAAGAA | 467 | 55 | 42 |
|  | Reverse | TCAACGTTCTTTGTCACAACC |  |  |  |
| *aacA-D* | Forward | TAATCCAAGAGCAATAAGGGC | 227 | 55 | 42 |
|  | Reverse | GCCACACTATCATAACCACTA |  |  |  |
| *tetK* | Forward | GTAGCGACAATAGGTAATAGT | 360 | 55 | 43 |
|  | Reverse | GTAGTGACAATAAACCTCCTA |  |  |  |
| *tetM* | Forward | AGTGGAGCGATTACAGAA | 158 | 55 | 42 |
|  | Reverse | CATATGTCCTGGCGTGTCTA |  |  |  |
| *linA* | Forward | GGTGGCTGGGGGGTAGATGTATTAACTGG | 323 | 57 | 43 |
|  | Reverse | GCTTCTTTTGAAATACATGGTATTTTTCGATC |  |  |  |
| *mecA* | Forward | AAAATCGATGGTAAAGGTTGG C | 532 | 56 | 43 |
|  | Reverse | AGTTCTGCAGTACCGGATTTGC |  |  |  |
| *fexA* | Forward | GTACTTGTAGGTGCAATTACGGCTGA | 1272 | 54 | 43 |
|  | Reverse | CGCATCTGAGTAGGACATAGCGTC |  |  |  |
| *cfr* | Forward | ATAGTGAGGAACGCAGCAAAT | 675 | 58 | 43 |
|  | Reverse | TCCAATGTCGCCTGTAGCA |  |  |  |
| *optrA* | Forward | GCTCTA GATTTCTCACCCAGATATGCC | 2150 | 50 | 43 |
|  | Reverse | CGGGATCCCGGCAAACTCAA AAGGTC |  |  |  |
| *vgaA* | Forward | CCAGAACTGCTATTAGCAGATGAA | 470 | 54 | 43 |
|  | Reverse | AAGTTCGTTTCTCTTTTCGACG |  |  |  |
| *vgaC* | Forward | ACTAACCAAGATACAGGACC | 734 | 52 | 42 |
|  | Reverse | TTATTGCTTGTCAGCCTTCC |  |  |  |
| *hla* | Forward | GGTTTAGCCTGGCCTTC | 534 | 58 | 44 |
|  | Reverse | CATCACGAACTCGTTCG |  |  |  |
| *hlb* | Forward | GTGCACTTACTGACAATAGTGC | 309 | 53 | 44 |
|  | Reverse | GTTGATGAGTAGCTACCTTCAGT |  |  |  |
| *hld* | Forward | GCCACTCATAACATATGGAA | 415 | 60 | 45 |
|  | Reverse | CAGATAAAGAGCTTTATACACACATTAC |  |  |  |
| *sea* | Forward | CATCCGAGTGAAACCCAAA | 560 | 56 | 44 |
|  | Reverse | GAAAAAAGTCTGAATTGCAGGGAACA |  |  |  |
| *seb* | Forward | ATTCTATTAAGGACACTAAGTTAGGGA | 404 | 57 | 44 |
|  | Reverse | ATCCCGTTTCATAAGGCGAGT |  |  |  |
| *sec* | Forward | GTAAAGTTACAGGTGGCAAAACTTG | 427 | 53 | 44 |
|  | Reverse | CATATCATACCAAAAAGTATTGCCGT |  |  |  |
| *sed* | Forward | AACTTAGGCATTCTGGCTCAC | 351 | 60 | 45 |
|  | Reverse | TCCCACTGTTCCATATCGTCA |  |  |  |
| *see* | Forward | ATAAATTGTTTCGGGTCGGTAAT | 577 | 57 | 44 |
|  | Reverse | AACCAGCCAACTAATGACAATGAT |  |  |  |
| *tst* | Forward | GCTTGCGACAACTGCTACAG | 599 | 54 | 45 |
|  | Reverse | TGGATCCGTCATTCATTGTTAT |  |  |  |
| *lukF* | Forward | CAACTCGAATTTTCAACAGGTACC | 466 | 56 | 44 |
|  | Reverse | CAGGCAGTCCATCTCCTG |  |  |  |
| *bap* | Forward | CTAGGAACTGCAATCTTAATCC | 492 | 53 | 44 |
|  | Reverse | TGGTAAAATCGCATGTCCAATTC |  |  |  |
| *icaA* | Forward | CCTAACTAACGAAAGGTAG | 348 | 59 | 44 |
|  | Reverse | AAGATATAGCGATAAGTGC |  |  |  |
| *icaD* | Forward | AAACGTAAGAGAGGTGG | 249 | 52 | 45 |
|  | Reverse | GGCAATATGATCAAGATA |  |  |  |
| *fnbA* | Forward | GTGAAGTTTTAGAAGGTGGAAAGATTAG | 643 | 57 | 44 |
|  | Reverse | GCTCTTGTAAGACCATTTTTCTTCAC |  |  |  |
| *fnbB* | Forward | GTAACAGCTAATGGTCGAATTGATACT | 524 | 54 | 44 |
|  | Reverse | CAAGTTCGATAGGAGTACTATGTTC |  |  |  |
| *clfA* | Forward | ATTGGCGTGGCTTCAGTGCT | 292 | 56 | 44 |
|  | Reverse | CGTTTCTTCCGTAGTTGCATTTG |  |  |  |
| *clfB* | Forward | GTAGCGACAATAGGTAATAGT | 360 | 55 | 45 |
|  | Reverse | GTAGTGACAATAAACCTCCTA |  |  |  |
